# Treatment outcomes following continuous miglustat therapy in patients with Niemann-Pick disease Type C: a final report of the NPC Registry

**DOI:** 10.1186/s13023-020-01363-2

**Published:** 2020-04-25

**Authors:** Marc C. Patterson, Eugen Mengel, Marie T. Vanier, Patrick Moneuse, Daniel Rosenberg, Mercedes Pineda

**Affiliations:** 1 Department of Neurology, Mayo Clinic, 200 first Street SW, Rochester, MN 55905 USA; 2 Villa Metabolica, University of Mainz, Mainz, Germany; 3 Present Address: SphinCS GmbH, Hochheim, Germany; 4 INSERM Unit 820, Faculté de Médecine RTH Laennec, Lyon, France; 5 Actelion Pharmaceuticals Ltd., A Janssen Pharmaceutical Company of Johnson & Johnson, Allschwil, Switzerland; 6 Institut Pediatric Hospital Sant Joan, Hospital Sant Joan de Déu, Passeig de Sant Joan de Deu, 2, 08950 Esplugues de Llobregat, Barcelona Spain

**Keywords:** Niemann-Pick disease type C, Miglustat, NPC disease registry, Treatment evaluation, Neurological symptoms, Safety, Tolerability, Natural history, Disease course

## Abstract

**Background:**

Niemann-Pick disease Type C (NP-C) is a rare, progressive neurodegenerative disorder characterized by progressive neurodegeneration and premature death. We report data at closure of the NPC Registry that describes the natural history, disease course and treatment experience of NP-C patients in a real-world setting.

**Methods:**

The NPC Registry was a prospective observational cohort study that ran between September 2009 and October 2017. Patients with a confirmed diagnosis of NP-C were enrolled regardless of treatment status. All patients underwent clinical assessments and medical care as determined by their physicians; data were collected through a secure internet-based portal.

**Results:**

At closure on October 19, 2017, 472 patients from 22 countries were enrolled in the NPC Registry. Mean (standard deviation) age at enrollment was 21.2 (15.0) years, and 51.9% of patients were male. First neurological symptom onset occurred during the early-infantile (< 2 years), late-infantile (2 to < 6 years), juvenile (6 to < 15 years), or adolescent/adult (≥ 15 years) periods in 13.5, 25.6, 31.8, and 29.1% of cases, respectively. The most frequent neurological manifestations prior to enrollment included ataxia (67.9%), vertical supranuclear gaze palsy (67.4%), dysarthria (64.7%), cognitive impairment (62.7%), dysphagia (49.1%), and dystonia (40.2%). During infancy, splenomegaly and hepatomegaly were frequent (*n* = 199/398 [50%] and *n* = 147/397 [37.0%], respectively) and persisted in most affected patients. Of the 472 enrolled patients, 241 were continuously treated with miglustat during the NPC Registry observation period, of whom 172 of these 241 patients were treated continuously for ≥12 months. A composite disability score that assesses impairment of ambulation, manipulation, language, and swallowing was highest in the early-infantile population and lowest in the adolescent/adult population. Among the continuous miglustat therapy population, 70.5% of patients had improved or had stable disease (at least 3 of the 4 domains having a decreased or unchanged score between enrollment and last follow-up). The NPC Registry did not identify any new safety signals associated with miglustat therapy.

**Conclusions:**

The profiles of clinical manifestations in the final NPC Registry dataset agreed with previous clinical descriptions. Miglustat therapy was associated with a stabilization of neurological manifestations in most patients. The safety and tolerability of miglustat therapy was consistent with previous reports.

## Background

Niemann-Pick disease Type C (NP-C) is a rare, progressive neurodegenerative disorder characterized by intracellular accumulation of cholesterol and complex lipids, such as sphingolipids and phospholipids, within the endosomal/lysosomal system [1, 2].

NP-C is caused by autosomal recessive mutations in either the *NPC1* or *NPC2* gene [3–5] and has an estimated incidence of between 1:100,000 and 1:120,000 live births, although this may be an underestimate [2, 6]. Patients with NP-C present at all ages with a heterogeneous spectrum of signs and symptoms across visceral, neurologic, and psychiatric domains, with characteristic symptomatology depending on the age of onset [2, 4, 7]. Disease onset in early infancy is characterized by visceral signs, such as liver and respiratory dysfunction, which in some cases can be rapidly progressive and fatal. Onset in childhood is characterized by a spectrum of visceral signs and neurological deficits, and onset in adults is characterized by a range of nonspecific neurological and psychiatric signs [1, 8–16]. The age at presentation of the first neurological manifestation is a predictor of disease progression and prognosis, with early-onset forms progressing more rapidly than late-onset forms [2, 4].

NP-C is invariably progressive, but reducing or halting progression of symptoms is key to optimal disease management [4, 7, 17]. Miglustat (Zavesca®, Actelion Pharmaceuticals Ltd.) is the only disease-specific therapy approved for NP-C.^1^ It has been shown to delay disease progression and to stabilize neurological symptoms in several randomized controlled clinical trials, observational studies, and long-term extension studies [7, 16, 18–20]. The NPC Registry was initiated in May 2009 as a post-approval commitment to the European Medicines Agency (EMA) following approval of a new indication for miglustat for the treatment of progressive neurological deterioration in adults and children with NP-C [1, 12]. The NPC Registry describes the natural history, disease course, clinical outcomes, and treatment experience in real-world clinical settings, and the data collected by the NPC Registry has proven invaluable to describe the natural history of the disease and treatment experience of patients [1, 12, 21].

Here we describe the characteristics of the patient population enrolled in the NPC Registry at closure in October 2017 and report the treatment experience of patients with NP-C who had received continuous miglustat therapy for more than 1 year during the observation period in the NPC Registry.

## Methods

### Study design and patients

The NPC Registry was an international, multicenter, prospective, observational cohort study in patients diagnosed with NP-C (EUPAS4622). All patients with a diagnosis of NP-C were eligible for inclusion in the NPC Registry regardless of their treatment. The methodology of the NPC Registry has previously been published [1, 12]. Data were collected via a secure internet-based portal, and written informed consent was obtained from all patients and/or their legal guardians before any clinical visit data were entered. Data entered to the NPC Registry included information routinely collected during clinical investigations for NP-C management as determined as appropriate by the treating physician.

These analyses include all patients in the NPC Registry from the commencement of enrollment in September 2009 up to database closure on 19th October 2017. The analysis to describe the patient treatment experience included all patients who received continuous miglustat therapy between the enrollment visit and their last follow-up visit. *Continuous miglustat therapy* was defined as the patient receiving miglustat for ≥90% of the observation period with no single period without receiving miglustat lasting > 28 days. A *miglustat switcher* was defined as a patient who had been treated with miglustat for < 90% of the observation time or had at least one period of > 28 days without miglustat treatment; switching does not imply that patients have switched from miglustat to other therapies. Patients were stratified based on the previously published age at neurological onset categories into early-infantile (< 2 years), late-infantile (2 to < 6 years), juvenile (6 to < 15 years), and adolescent/adult (≥ 15 years) populations [2, 4].

Due to small sample sizes, patients who switched to other therapies and those who were not treated with miglustat were not included in analyses of treatment evaluation.

### Assessments of disease status and progression

An assessment of disability status was performed using a previously described modified NP-C disability scale [7, 11]; composite disability scores were calculated as the average of the scores from each of the 4 individual domains (ambulation, manipulation, language, swallowing) of the disability scale. Scores for each domain ranged from 0 with no disability, to 1 being the most affected (Table 1). The extent of change in each of the 4 domains of the disability scale was evaluated from enrollment to the last follow-up visit as improved (decrease in score), stable (no change in score), or progressed (increase in score). Overall neurological progression was considered improved or stable if at least 3 of the 4 individual domain scores were improved or stable during the observation period. The annual progression rate of the composite disability score was computed as the change in disability scale score from enrollment to the last follow-up visit divided by the time from enrollment to the last follow-up visit.

**Table 1:** Modified disability scale

Functional areas	Score
**Ambulation**	
Normal	0
Autonomous ataxic gait	0.25
Outdoor assisted ambulation	0.50
Indoor assisted ambulation	0.75
Wheelchair-bound	1
**Manipulation**	
Normal	0
Slight dysmetria/dystonia	0.33
Mild dysmetria/dystonia	0.67
Severe dysmetria/dystonia	1
**Language**	
Normal	0
Mild dysarthria	0.25
Severe dysarthria	0.50
Non-verbal communication	0.75
Absence of communication	1
**Swallowing**	
Normal	0
Occasional dysphagia	0.33
Daily dysphagia	0.67
Nasogastric tube or gastric button feeding	1

Data related to biomarkers of disease progression were not available from the NPC Registry.

Safety-relevant information, that included adverse drug reactions and adverse events, was collected as part of the NPC Registry.

### Statistical analyses

Descriptive analyses were performed on the whole enrolled NPC Registry population and the continuous miglustat therapy population. Clinical disability assessments as a reflection of clinical outcomes were further described in the continuous miglustat therapy population and in patients continuously treated with miglustat for ≥ 12 months. Safety-relevant data are descriptively summarized for the continuous miglustat therapy population only.

Analyzes of data at enrollment are purely descriptive in nature. Continuous variables are summarized using descriptive statistics including mean, standard deviation (SD), median, range and 95% confidence interval (CI) of the mean. Categorical variables are summarized using counts and percentages. As this study is a registry, this analysis is of observational data with all summary statistics and percentages calculated relative to number of patients with available data. Denominators for analysis were the numbers of patients with the corresponding data available; different parameters may have different denominators.

## Results

### Demographics and patient characteristics

#### Enrolled population

At database closure, 472 patients from 22 countries were enrolled in the NPC Registry (see Additional file 1), with a similar proportion of males (*n* = 245; 51.9%) and females (*n* = 227; 48.1%). The mean (standard deviation [SD]) age at enrollment was 21.2 (15.0) years, with the majority (*n* = 291/470; 61.9%) aged between 10 and 40 years; 47.7% of enrolled patients were aged < 18 years. Patients with known age at onset of neurological symptoms (*n* = 422) were categorized as early-infantile (*n* = 57; 13.5%), late-infantile (*n* = 108; 25.6%), juvenile, (*n* = 134; 31.8%), or adolescent/adult (*n* = 123; 29.1%) onset (Table 2). A diagnostic delay was apparent in each of these age at neurological onset categories, with a mean delay between the appearance of first neurological symptoms and a confirmed diagnosis of NP-C of 2.5 years in early-infantile, 4.3 years in late-infantile, 6.2 years in juvenile, and 6.3 years in adolescent/adult patients (Table 2).

**Table 2:** Demographics and characteristics of patients enrolled in the NPC Registry (*N* = 472)

	Patient characteristics of overall population (*N* = 472)	Patients with age at neurological onset data			
		Early-infantile (< 2 years)	Late-infantile (2 to < 6 years)	Juvenile (6 to < 15 years)	Adolescent/adult onset (≥ 15 years)
**Male: female, n (%**^**a**^**)**	245 (51.9): 227 (48.1)	–	–	–	–
**Age at enrollment**					
*n*	470	–	–	–	–
Mean (SD), years	21.2 (15.0)	–	–	–	–
Median (range), years	19.0 (0.2–71.8)	–	–	–	–
**Age at onset of neurological symptoms**					
*n* (%^a^)	422 (100.0)	57 (13.5)	108 (25.6)	134 (31.8)	123 (29.1)
Mean (SD), years	12.3 (11.8)	0.8 (0.7)	4.0 (1.2)	10.1 (2.6)	27.2 (11.0)
Median (range), years	8.8 (0.0–71.8)	0.8 (0.0–2.0)	4.0 (2.0–6.0)	10.0 (6.0–15.0)	25.0 (15.0–71.8)
**Age at diagnosis**					
*n* (%^a^)	292 (100.0)	37 (12.7)	84 (28.8)	87 (29.8)	84 (28.8)
Mean (SD), years	–	3.3 (4.6)	8.3 (7.4)	16.3 (8.6)	33.5 (12.4)
Median (range), years	–	1.6 (0.1–21.4)	6.7 (0.1–33.1)	13.9 (2.9–56.3)	31.8 (14.4–69.8)

*SD* Standard deviation

^a^Percentage based on patients with data (excluding missing responses)

The most frequent neurological manifestations reported in the medical history prior to enrollment include ataxia (*n* = 304/448; 67.9%), vertical supranuclear gaze palsy (*n* = 302/448; 67.4%), dysarthria (*n* = 290/448; 64.7%), cognitive impairment (*n* = 281/448; 62.7%), dysphagia (*n* = 220/448; 49.1%) and dystonia (*n* = 180/448; 40.2%) (Table 3). A number of other neurological manifestations were less frequently present within the population (Table 3). During infancy, splenomegaly and hepatomegaly were frequent (*n* = 199/398; 50.0%, and *n* = 147/397; 37.0%, respectively; Table 3) and persisted in most of the afflicted patients. Most patients with splenomegaly during infancy also presented with hepatomegaly (*n* = 139/199; 69.8%).

**Table 3:** History of NP-C manifestations for all patients in the NPC Registry prior to enrollment

Manifestation	*N*	Overall patients n (%)
**Neurological manifestations**		
Ataxia	448	304 (67.9)
Vertical supranuclear gaze palsy	448	302 (67.4)
Dysarthria	448	290 (64.7)
Cognitive impairment/learning difficulties/school failure	448	281 (62.7)
Dysphagia	448	220 (49.1)
Dystonia	448	180 (40.2)
Seizures	448	115 (25.7)
Clumsiness	448	92 (20.5)
Cataplexy	448	87 (19.4)
Behavioral disturbance	448	63 (14.1)
Psychiatric manifestations	448	50 (11.2)
Sleep disturbance	448	36 (8.0)
Difficulties stepping down	448	31 (6.9)
Horizontal gaze palsy	448	22 (4.9)
Other	448	21 (4.7)
**Visceral manifestations in infancy**		
Splenomegaly	398	199 (50.0)
Hepatomegaly	397	147 (37.0)
Pulmonary infiltration	448	24 (5.4)

*N* Number of enrolled patients with evaluable data

#### Continuous miglustat therapy population

**Fig. 1:**
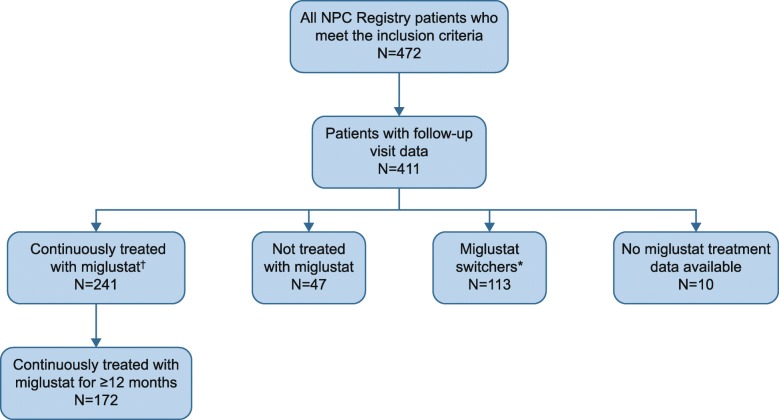
Patient flow. * Patients who had periods of treatment and nontreatment and had been treated with miglustat for < 90% of the observation time or had one period without miglustat lasting more than 28 days. † Among patients continuously treated with miglustat, 216/241 had received miglustat prior to enrollment for a varying period

Of the 472 enrolled patients, 241 were continuously treated with miglustat during the NPC Registry observation period, of whom 172 were continuously treated with miglustat for ≥ 12 months (Fig. 1). A further 47 patients were not treated with miglustat, and 113 were miglustat switchers; no data on miglustat treatment were available for 10 patients. Of the 241 patients who had continuous miglustat therapy, the majority (*n* = 216) had received miglustat prior to enrollment with a mean (SD; median [range]) duration of treatment of 2.06 (2.07; 1.39 [0.00–9.97]) years prior to enrollment. Mean (SD; median [range]) duration of treatment during the observation period of the NPC Registry was 3.27 (1.95; 3.29 [0.11–7.62]) years, which does not include treatment exposure prior to the observation period. Within the continuous miglustat therapy population, the mean age (SD) at neurological onset was 11.2 (10.2) years, at diagnosis was 15.0 (11.9) years (Table 4), and at enrollment was 20.0 (12.4) years.

**Table 4:** Demographics and disease characteristics of patients continuously treated with miglustat in the NPC Registry by age at neurological onset category (*N* = 241)

	Patients continuously treated with miglustat	Patients with age at neurological onset data^**a**^			
		Early-infantile (< 2 years)	Late-infantile (2 to < 6 years)	Juvenile (6 to < 15 years)	Adolescent/adult onset (≥ 15 years)
**Age at onset of neurological manifestations**					
*n* (%^a^)	224 (100.0)	21 (9.4)	66 (29.5)	81 (36.2)	56 (25.0)
Mean (SD), years	11.2 (10.2)	0.9 (0.6)	4.0 (1.3)	9.9 (2.7)	25.5 (9.7)
Median (range), years	8.1 (0.0–54.8)	1.0 (0.0–2.0)	3.9 (2.0–6.0)	9.6 (6.1–15.0)	22.1 (15.3–54.8)
**Age at diagnosis**					
*n* (%^a^)	171 (100.0)	17 (10.4)	53 (32.5)	56 (34.4)	37 (22.7)
Mean (SD), years	15.0 (11.9)	4.6 (5.5)	8.1 (6.9)	14.8 (6.4)	30.5 (11.5)
Median (range), years	12.7 (0.1–68.8)	2.5 (0.1–21.4)	7.3 (0.1–28.2)	13.7 (2.9–40.2)	28.1 (14.4–68.8)
**Composite disability score at enrollment**					
*n* (%^a^)	221 (100.0)	19 (8.6)	60 (27.1)	76 (34.4)	53 (24.0)
Mean (SD)	0.38 (0.26)	0.59 (0.35)	0.34 (0.28)	0.43 (0.25)	0.32 (0.16)
Median (range)	0.29 (0.00–1.00)	0.63 (0.00–1.00)	0.29 (0.00–1.00)	0.43 (0.00–1.00)	0.29 (0.00–0.81)
95% CI of mean	0.35–0.42	0.42–0.76	0.27–0.42	0.38–0.49	0.28–0.37
**Composite disability score at last follow-up**					
*n* (%^a^)	235 (100.0)	19 (8.1)	63 (26.8)	80 (34.0)	56 (23.8)
Mean (SD)	0.48 (0.29)	0.70 (0.34)	0.51 (0.32)	0.49 (0.26)	0.39 (0.23)
Median (range)	0.44 (0.00–1.00)	0.94 (0.00–1.00)	0.50 (0.00–1.00)	0.49 (0.00–1.00)	0.31 (0.00–0.94)
95% CI of mean	0.44–0.51	0.54–0.87	0.43–0.59	0.43–0.55	0.33–0.45

*CI* Confidence interval, *N* Number of enrolled patients with evaluable data, *SD* Standard deviation

^a^Percentages based on patients with available data; for some patients this information is unknown or missing

During infancy, 54.5% (*n* = 109/200) and 36.0% (*n* = 73/203) of patients continuously treated with miglustat presented with splenomegaly and hepatomegaly, respectively (Table 5), and most patients with splenomegaly also presented with hepatomegaly (*n* = 70/109; 64.2%). Of 227 patients with available data, 71.4% presented with ataxia (*n* = 162), 70.5% with vertical supranuclear gaze palsy (*n* = 160), 68.7% with dysarthria (*n* = 156), 59.9% with cognitive impairment (*n* = 136), 48.9% with dysphagia (*n* = 111), and 42.7% with dystonia (*n* = 97) prior to enrollment (Table 5). Of patients continuously treated with miglustat with data at the time of enrollment, 92/113 (81.4%) had neurological abnormalities, 46/191 (24.1%) had psychiatric manifestations, 50/113 (44.2%) had behavioral signs, and 17/197 (8.6) had respiratory tract abnormalities (Table 5).

**Table 5:** History of NP-C manifestations for patients continuously treated with miglustat

Manifestation	*N*	*n* (%)
**Neurological manifestations prior to enrollment**		
Ataxia	227	162 (71.4)
Vertical supranuclear gaze palsy	227	160 (70.5)
Dysarthria	227	156 (68.7)
Cognitive impairment	227	136 (59.9)
Dysphagia	227	111 (48.9)
Dystonia	227	97 (42.7)
Seizures	227	70 (30.8)
Clumsiness	227	50 (22.0)
Cataplexy	227	48 (21.1)
Behavioral disturbance	227	33 (14.5)
Psychiatric manifestation	227	26 (11.5)
**Visceral manifestations during infancy**		
Splenomegaly	200	109 (54.5)
Hepatomegaly	203	73 (36.0)
**General examination abnormalities at enrollment**		
Neurological, any	113	92 (81.4)
Behavioral	113	50 (44.2)
Musculoskeletal	190	46 (24.2)
Psychiatric	191	46 (24.1)
Gastrointestinal	194	25 (12.9)
Sleep, any	107	17 (15.9)
Respiratory tract	197	17 (8.6)
Genitourinary	182	13 (7.1)
Head/neck	186	11 (5.9)
Skin	185	9 (4.9)
Cardiovascular	198	5 (2.5)
Lymphatic	175	1 (0.6)

*NP-C* Niemann-Pick disease type C

### Disease progression in the continuous miglustat therapy population

At enrollment, the mean (SD) composite disability score for the continuous miglustat therapy population (*n* = 221) was 0.38 (0.26). It was highest in the early-infantile group (0.59 [0.35]) and lowest in the adolescent/adult group (0.32 [0.16]). At last follow-up the mean (SD) composite disability score was 0.48 (0.29). It was highest in the early-infantile population (0.70 [0.34]), and lowest in the adult/adolescent population (0.39 [0.23]) (Table 4).

**Fig. 2:**
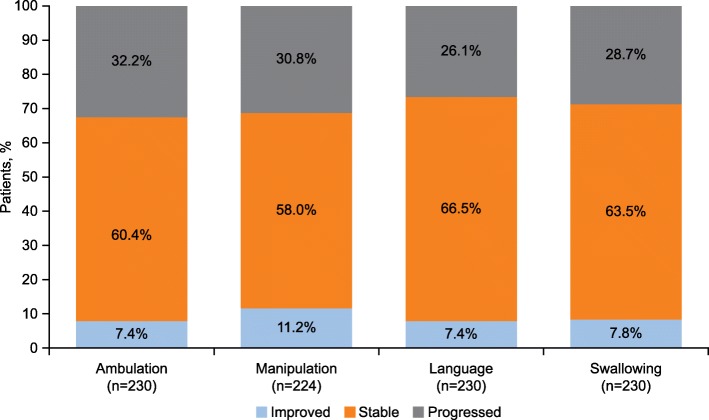
Change in disability scale scores from enrollment to the last follow-up visit in patients continuously treated with miglustat

Overall, 70.5% (*n* = 153/217) of the continuous miglustat therapy population had improved or stable disease, with at least 3 of the 4 domains having a decreased score or remaining unchanged between enrollment and last follow-up. Decreased scores, indicative of lessened severity of symptoms, were observed in a small proportion of patients across all domains. Stable scores were observed in the majority of patients in all domains (Fig. 2). Stable or decreased scores were observed for all domains: ambulation (*n* = 156/230; 67.8%), manipulation (*n* = 155/224; 69.2%), language (*n* = 170/230; 73.9%), and swallowing (*n* = 164/230; 71.3%) (Fig. 2).

**Fig. 3:**
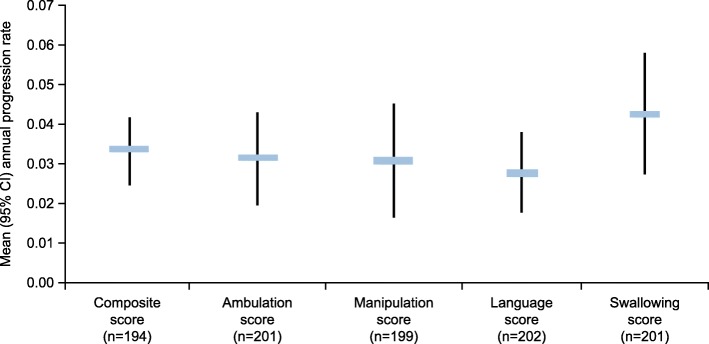
Annual progression rate of disability scale scores from enrollment to the last follow-up visit in patients continuously treated with miglustat. CI, confidence interval

The mean annual progression rate of the composite disability score in the entire continuous miglustat therapy population (*n* = 194/210) was 0.034 (95% confidence interval [CI] 0.025–0.042) after a mean (SD) observation period of 3.67 (1.77) years. Of the individual domains, swallowing had the highest mean annual progression rate (0.043; 95% CI 0.028–0.058); annual progression of the other domains was slower (ambulation [0.032; 95% CI 0.020–0.043]; manipulation [0.031; 95% CI 0.017–0.045]; language [0.028; 95% CI 0.018–0.038]) (Fig. 3). In patients who received continuous miglustat therapy for ≥ 12 months (*n* = 160/172), the mean annual progression rate of the composite disability score was similar to that of the entire continuous miglustat therapy population (0.036; 95% CI 0.025–0.047), as were the scores for the individual domains (ambulation [0.035; 95% CI 0.021–0.049], manipulation [0.033; 95% CI 0.017–0.048], language [0.028; 95% CI 0.016–0.040], swallowing [0.049; 95% CI 0.029–0.070]).

### Safety in the continuous miglustat therapy population

In this final output from the NPC Registry, no new safety concerns were identified; the safety-relevant information obtained from the NPC Registry was consistent with the known safety profile of miglustat in NP-C (Table 6).

**Table 6:** Safety information for patients continuously treated with miglustat before miglustat initiation and during NPC Registry follow-up (*N* = 241)

Safety information/event	Time period	Patient, *N*	Patient with events, n (%^e^)
Seizures	Pretreatment: present	241	81 (33.6)
	During follow-up: new or worsened^d^	103	48 (46.6)
Thrombocytopenia^a^	Pretreatment: present	241	49 (20.3)
	During follow-up: present	211	109 (51.7)
Tremor	Pretreatment: present	241	86 (35.7)
	During follow-up: new or worsened^d^	241	37 (15.4)
Neuropathy	Pretreatment: present	240	18 (7.5)
	During follow-up: new or worsened^d^	241	19 (7.9)
Chronic diarrhea^b^	Pretreatment: present	241	11 (4.6)
	During follow-up: new or worsened^d^	241	27 (11.2)
Other^c^	Pretreatment: present	231	13 (5.6)

^a^101 patients had mild thrombocytopenia (101–150 × 10^9^/L) and 32 had moderate thrombocytopenia (51–100 × 10^9^/L)

^b^Diarrhea lasting > 3 months

^c^Any other possibly related adverse event not considered as thrombocytopenia, neuropathy, seizure, tremor, or gastrointestinal-related event

^d^Event occurred at least once during follow-up

^e^Percentages based on patients with available data; for some patients this information is unknown or missing

Known safety/tolerability considerations associated with miglustat, including chronic diarrhea, thrombocytopenia, and seizures, were frequently reported in the overall population. Chronic diarrhea (i.e., diarrhea lasting > 3 months) occurred in 11.2% (*n* = 27/241) of patients during the observation period and in 4.6% (*n* = 11/241) of patients before treatment. Seizures were present in a total of 33.6% (*n* = 81/241) of patients before miglustat therapy, and 46.6% of patients with available data (*n* = 48/103) had a new occurrence or worsened seizures during follow-up. Thrombocytopenia was recorded in 20.3% (*n* = 49/241) of patients at enrollment and in 51.7% (*n* = 109/211) of patients during follow-up; almost all cases of thrombocytopenia were mild or moderate. Amongst patients with thrombocytopenia it is likely related to splenomegaly, which was present in 71.1% of evaluable patients at enrollment; 72.7% of evaluable patients during follow-up. Tremor was present before miglustat initiation in 35.7% (*n* = 86/241) of patients, and newly occurring or worsened tremor was reported for 37/241 (15.4%) patients during follow-up. Neuropathy was present in 7.5% (*n* = 18/240) of patients before miglustat initiation; newly occurring or worsened neuropathy was reported for 19/241 (7.9%) patients during follow-up.

The most common reasons for discontinuation of miglustat therapy were death and progression of NP-C disease (Supplementary Table 2).

## Discussion

Here, we present the characteristics of the 472 patients enrolled in the NPC Registry at database closure, expanding on previous reports of the NPC Registry dataset [1, 12]. The NPC Registry is the largest repository of data for patients with this ultra-rare disease and allows reporting of the natural history, disease course, clinical outcomes, and treatment experience of patients with NP-C in real-world clinical settings. The description of NP-C from these data is consistent with previous reports of the natural history and disease course [1, 15, 16].

Since the most recent previous description of the entire enrolled population of the NPC Registry [1], the number of patients has greatly increased from 163 in the 2013 report to 472 in the present report, providing a greater wealth of data for all age at neurological onset categories. The proportion of patients within each of the age at neurological onset categories has remained relatively consistent (early-infantile, [2013] 11% vs [present] 13.5%; late-infantile, 31% vs 25.6%; juvenile, 31% vs 31.8%; adolescent/adult, 27% vs 29.1%) [1]. The present data, reporting the age at onset of neurological symptoms, the age at diagnosis by age of neurological onset, and the percentages of patients in which the individual cardinal symptoms of NP-C manifestations occur, are also consistent between the two reports [1]. The proportions of patients who presented at enrollment with the characteristically wide range of presenting visceral, neurological, and psychiatric symptoms are consistent with current understanding of the disease [1, 2, 8, 10, 15, 16]. Neurological signs such as ataxia, vertical supranuclear gaze palsy, dysarthria, and cognitive impairment were the most common, being identified in around two-thirds of patients. Splenomegaly is relatively common and is usually accompanied by hepatomegaly in this population. It should be noted that the frequency of these findings may be an underestimate, as some signs, such as the vertical supranuclear gaze palsy, are often not recognized by less-experienced clinicians. Similarly, organomegaly may be missed in those patients who undergo only a physical examination and can often be detected only through ultrasound or other imaging modalities.

We also present longitudinal analysis of functional disability in patients who were treated continuously with miglustat for at least 1 year. Functional disability, measured as a composite of deficits in ambulation, manipulation, language, and swallowing, was higher for patients in whom neurological symptoms manifest at a younger age than in patients who are older when neurological signs first appear. This is consistent with our understanding of NP-C as a disease, which has more severe presentation and rapid progression of symptoms in patients whose neurological manifestations appear at a younger age [2]. Functional disability was stable or improved in a majority (70.5%) of patients who had received continuous miglustat therapy and is consistent with previously reported findings using this same disability assessment method [7]. These findings are also consistent with earlier analyses of the NPC Registry, which reported a mean annual composite disability score increase of 0.038 (95% CI 0.018, 0.059), compared with 0.036 (95% CI 0.025,0.047) [12]. More broadly, these functional disability findings complement the wealth of data that support the use of miglustat as an effective therapy for the stabilization of the neurological manifestations of NP-C [16, 18–20, 22, 23].

A majority of the patients were continuously treated with miglustat for at least 12 months, which is sufficient to observe treatment benefits [4, 24]. Most of these patients had been receiving miglustat therapy prior to their enrollment to the NPC Registry; some had been receiving miglustat for several years. In line with the product label for miglustat, most subjects who have progressive neurological manifestations will have been enrolled into the miglustat treated cohort, apart from a very small number of subjects who did not or could not commence miglustat treatment. The remainder of the untreated cohort are those subjects in whom NP-C has not presented as the progressive neurological form. Consequently, the disease characteristics of the two cohorts are not easily comparable, confounding any side-by-side comparisons of treatment efficacy. A challenge also remains to account for confounding by indication, as reasons for initiation of miglustat treatment were not collected, as well as other unmeasured confounders. Further limiting these comparisons is the imbalance in numbers between cohorts, e.g. only 47 subjects for the not-treated cohort. For these reasons, it is not possible to compare the disease course and prognosis between those patients who were treated or untreated with miglustat.

No new safety findings were observed since the start of the NPC Registry. The tolerability of miglustat treatment within the NPC Registry dataset was consistent with observations from previous clinical and observational studies [18–20] and with those reported in previous descriptions of the NPC Registry [1, 12]. Diarrhea was relatively common but is known to be associated with miglustat treatment; simple dietary modifications and up-titration of drug dose can alleviate these symptoms in many patients [4, 25, 26]. Although the incidence of both tremor and seizures increased during the follow-up period, this is likely reflective of the worsening of neurological manifestations as part of the natural disease course. No increase in neuropathy was reported during the observation period.

These observations from the final NPC Registry population of 472 patients represent the largest database of patients with NP-C reported to date. As with any disease registry data, caution must be taken with their interpretation, as the integrity of any disease registry database relies on the accurate entry of patient information by the treating physicians and the staff at each participating center; this may explain some outlier data, for which age at neurological onset may have been recalled or entered incorrectly. However, due to the wealth of data available in this dataset on such a large number of patients with NP-C, these findings provide a valuable contribution to our existing knowledge of the disease and patient characteristics.

## Conclusions

In addition to its important role in post-approval monitoring, the NPC Registry has provided an unparalleled wealth of data on NP-C. This final report of the NPC Registry database provides the largest ever NP-C-specific clinical dataset, which confirms and strengthens our understanding of the natural history and disease course of NP-C and supports previous findings of the effectiveness of miglustat as a disease-modifying therapy that can stabilize progression of the disease.

## Supplementary information

**Additional file 1: Supplementary Table 1.** Countries from which patients were enrolled to the NPC Registry up until database closure. **Additional file 1: Supplementary Table 2.** Reasons for discontinuation of miglustat treatment.

## Data Availability

The data sharing policy of the Sponsor is available at https://www.janssen.com/clinical-trials/transparency. As noted on this site, requests for access to the study data can be submitted through Yale Open Data Access (YODA) Project site at http://yoda.yale.edu.

## Abbreviations

CI: Confidence interval
EMA: European Medicines Agency
NP-C: Niemann-Pick disease type C
SD: Standard deviation

## Footnotes

1 Miglustat is indicated for the treatment of progressive neurologic manifestations in adult and pediatric patients with NP-C. Miglustat is approved for NP-C under the brand name Zavesca® in Argentina, Australia, Brazil, Canada, Chile, China, Colombia, Ecuador, European Union/European Economic Area, Iceland, Liechtenstein, Iran, Israel, Mexico, New Zealand, Norway, Palestine, Panama, Peru, Russia, South Korea, Thailand, Turkey, and Venezuela. In Japan, miglustat is approved for NP-C under the brand name Brazaves®. Miglustat is not approved for the treatment of NP-C in the US and Taiwan.
