# Supplementary material for: Treatment outcomes following continuous miglustat therapy in patients with Niemann-Pick disease Type C: a final report of the NPC Registry
Source: Orphanet J Rare Dis. 2020 Apr 25;15:104. doi: 10.1186/s13023-020-01363-2 (PMC7183679; doi:10.1186/s13023-020-01363-2)
Supplement: Supplementary file 1 — Additional file 1: Supplementary Table 1. Countries from which patients were enrolled to the NPC Registry up until database closure. Additional file 1: Supplementary Table 2. Reasons for discontinuation of miglustat treatment. [file 13023_2020_1363_MOESM1_ESM.docx]

**Supplementary Table 1.** Countries from which patients were enrolled to the NPC Registry up until database closure

| **Country** | **Number of centers** | **n (%)** |
| --- | --- | --- |
| N | 112 | 472 (100.0) |
| Australia | 2 | 10 (2.1) |
| Austria | 2 | 3 (0.6) |
| Brazil | 7 | 40 (8.5) |
| Bulgaria | 1 | 12 (2.5) |
| Canada | 7 | 16 (3.4) |
| China | 1 | 3 (0.6) |
| Czech Republic | 2 | 19 (4.0) |
| France | 12 | 48 (10.2) |
| Germany | 20 | 89 (18.9) |
| Greece | 4 | 6 (1.3) |
| Italy | 10 | 42 (8.9) |
| Netherlands | 5 | 21 (4.4) |
| Norway | 1 | 1 (0.2) |
| Poland | 5 | 16 (3.4) |
| Portugal | 3 | 4 (0.8) |
| Russia | 2 | 16 (3.4) |
| Slovak Republic | 2 | 12 (2.5) |
| Slovenia | 1 | 2 (0.4) |
| Spain | 9 | 20 (4.2) |
| Sweden | 4 | 5 (1.1) |
| Switzerland | 2 | 14 (3.0) |
| United Kingdom | 10 | 73 (15.5) |

**Supplementary Table 2.** Reasons for discontinuation of miglustat treatment

| **Reason for discontinuation** | **n (%)^a^** |
| --- | --- |
| Patients treated ≥1 with miglustat and with 1 follow-up visit | 364 |
| Patients who discontinued | 95 |
| Any reason to discontinue^b^ | 139 (100) |
| Death | 42 (30.2) |
| Pregnancy | 1 (0.7) |
| GI disorder | 18 (12.9) |
| Other adverse event considered possibly related to miglustat | 12 (8.6) |
| Progression of NP-C disease | 22 (15.8) |
| Lost to follow-up | 1 (0.7) |
| Non-medical reason | 19 (13.7) |
| Unknown | 24 (17.3) |

^a^ Percentage based on patients with data (excluding missing responses)
^b^ Some patients discontinued more than once
